# Dental evolutionary rates and its implications for the Neanderthal–modern human divergence

**DOI:** 10.1126/sciadv.aaw1268

**Published:** 2019-05-15

**Authors:** Aida Gómez-Robles

**Affiliations:** 1 Department of Anthropology, University College London, 14 Taviton St., London WC1E 0BW, UK.; 2 Department of Genetics, Evolution and Environment, University College London, Gower St., London WC1E 6BT, UK.; 3 Department of Life Sciences, Natural History Museum, Cromwell Road, London SW7 5BD, UK. Email: a.gomez-robles@ucl.ac.uk

## Abstract

The origin of Neanderthal and modern human lineages is a matter of intense debate. DNA analyses have generally indicated that both lineages diverged during the middle period of the Middle Pleistocene, an inferred time that has strongly influenced interpretations of the hominin fossil record. This divergence time, however, is not compatible with the anatomical and genetic Neanderthal affinities observed in Middle Pleistocene hominins from Sima de los Huesos (Spain), which are dated to 430 thousand years (ka) ago. Drawing on quantitative analyses of dental evolutionary rates and Bayesian analyses of hominin phylogenetic relationships, I show that any divergence time between Neanderthals and modern humans younger than 800 ka ago would have entailed unexpectedly rapid dental evolution in early Neanderthals from Sima de los Huesos. These results support a pre–800 ka last common ancestor for Neanderthals and modern humans unless hitherto unexplained mechanisms sped up dental evolution in early Neanderthals.

## Teaser

The analysis of dental evolutionary rates indicates that Neanderthals and modern humans diverged at least 800,000 years ago.

## INTRODUCTION

The timing and the identity of the last common ancestor (LCA) of *Homo neanderthalensis* and *Homo sapiens* (referred to as Neanderthals and modern humans hereafter) are intensely debated issues (*1*–*5*). Studies of ancient DNA (aDNA) have generally pointed to a divergence time of ca. 400 thousand years (ka) ago (*6*), which has found support in some quantitative studies of cranial variation (*7*). In addition, typically discussed evolutionary scenarios tend to assume that at least some Middle Pleistocene hominins dated to 600 to 400 ka ago, or even younger, were part of the last common ancestral species to Neanderthals and modern humans [reviewed in (*8*)]. Multiple anatomical studies of the fossil evidence, however, have indicated that some Middle Pleistocene European hominins, particularly those belonging to the Sima de los Huesos (SH) sample, show clear affinities with Neanderthals (*9*–*11*). After some conflicting results regarding the geological age of the SH hominins (*12*, *13*), this collection is now securely dated to 430 ka ago (*14*), an age that is confirmed by the analyses of the length of its mitochondrial DNA (mtDNA) branch (*15*). In addition, recent analyses of the nuclear DNA (nDNA) of this population have demonstrated an evolutionary affinity of SH hominins with classic Neanderthals (*16*), thus making the divergence between Neanderthals and modern humans necessarily older than the age of the SH fossils. Some recent studies reflect these new findings and favor an earlier age for this LCA of 550 to 765 ka (*17*) based on more recent estimates of the human mutation rate (*16*). Divergence times inferred from genomic data are highly dependent on mutation rate and generation time estimates, which are still debated (*18*). Small variations of these parameters can result in very different estimates of the divergence time between two species. If these nuances are not considered, then a strict read of the values provided by aDNA analyses can give rise to radically different interpretations of the fossil record, which can even be incompatible with the affinities inferred from the anatomical evidence.

**Fig. 1:**
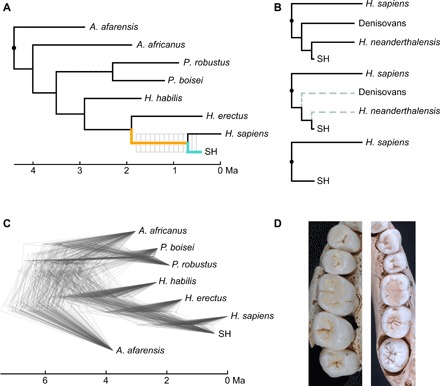
Phylogenetic scenarios and SH dental morphology. (**A**) Hominin phylogeny used in the analysis of evolutionary rates (phylogeny-1). The SH branch is represented in teal, and the LCA branch in orange, which are the colors used to represent the evolutionary rates on these two branches in Figs. 4 and 5 and fig. S5. Gray lines represent the different divergence times that have been evaluated. (**B**) Transformation of the Neanderthal-Denisovan-SH lineage into the SH lineage. (**C**) Densitree showing a randomly selected sample of 100 phylogenies [of the total sample of 60,000 phylogenies generated by Dembo and colleagues’ Bayesian analysis of hominin phylogenetic relationships (*20*)]. Dembo’s original trees have been pruned to preserve only the species for which dental data are available. The length of the Neanderthal branch has been shortened to reflect the age of the SH branch. (**D**) Upper and lower postcanine dentition of one representative SH individual (upper dentition is represented on the left). Photo credit: A. Muela, photographs taken at Institute of Health Carlos III.

The closer evolutionary affinity of SH with Neanderthals than with modern humans indicates that SH hominins diverged from the modern human lineage at the same point as classic Neanderthals did. Therefore, the genetic affinities, geological age, and morphological variation of SH hominins can be used to infer the timing of the Neanderthal–modern human divergence. Recent studies of hominin variation have demonstrated that, unlike other traits, postcanine dental shape as described through geometric morphometric datasets (fig. S1) has evolved neutrally and at extremely homogenous rates in all hominin lineages (*19*). This observation was used in the present study to infer the time at which Neanderthals and modern humans should have diverged to maintain the evolutionary rate for dental shape of the phylogenetic branch leading to SH hominins within the same range of variation observed in the other hominin species (tables S1 and S2). Dental shape in SH hominins is unexpectedly derived toward the Neanderthal condition, both in the expression of Neanderthal discrete features (*9*) and in its extreme degree of postcanine structural reduction in the number and size of cusps (fig. S2) (*11*). The dental shape of SH hominins is so derived that it is not representative of other Neanderthal populations. However, this does not affect the design of this study. Even if SH hominins do not show the average dental shape observed in later classic Neanderthals, their highly derived dentitions must have evolved from the same ancestral shape as classic Neanderthals did and over the period of time that separates the SH hominins from the Neanderthal–modern human LCA (see Fig. 1). The homogeneity of evolutionary rates for dental shape stands out in stark contrast with the much more heterogenous scenario observed for dental size, for which different rates are observed at different branches of the hominin phylogeny (*19*).

To account for the lack of consensus on hominin phylogenetic relationships, analyses were based on two different phylogenetic frameworks (fig. S3) (*19*, *20*). The first one (phylogeny-1) is the phylogenetic tree used in a previous study of hominin evolutionary rates (*19*), which is based on the first and last appearance dates of those hominin species for which shape data for all posterior teeth were available. The second phylogeny (phylogeny-2) is the maximum clade credibility (MCC) tree calculated by Dembo and colleagues (*20*, *21*) as part of their Bayesian analysis of hominin phylogenetic relationships. This phylogeny was pruned to include only the species for which dental data were available. In those two phylogenies, the age of the Neanderthal–modern human LCA was changed from 500 ka, which is right below the lowest bound of the interval suggested by the most recent molecular analyses (*16*, *17*, *22*), to the age of the subtending node at 100-ka intervals (Fig. 1). Uncertainty about hominin phylogenic relationships and branch lengths was explicitly addressed by estimating evolutionary rates over a sample of 100 trees. This sample of trees was randomly selected out of a sample of 60,000 trees generated through the Bayesian analysis of the hominin phylogeny (*20*, *21*) (Fig. 1). Denisovans (*23*), who diverged from classic Neanderthals after the Neanderthal–modern human divergence but before the age of SH fossils (*16*), were not incorporated into these analyses because very scarce phenotypic data are available for this group. However, considering their evolutionary relationships (*24*), Denisovans, as SH hominins, can be considered part of the Neanderthal lineage in the broad sense or *H. neanderthalensis sensu lato* (Fig. 1).

The used methodological approach consisted of a three-step process that included calculating ancestral values using a multiple variance Brownian motion (mvBM) approach (*25*), calculating the amount of change per branch as the difference between descendant and ancestral morphologies, and comparing these values with those obtained when simulating evolution at a constant rate across all branches of the hominin phylogeny (*19*). The major advantage of this approach is that it specifically and quantitatively accounts for the possibility that the LCA of Neanderthals and modern humans (or of any other two species) was not intermediate in morphology between both daughter species but more similar to Neanderthals. This is a scenario that has been recently suggested to explain the presence of derived Neanderthal features in the SH sample (*4*) and even in earlier European hominins (*26*), but that has not been formally tested yet.

## RESULTS

**Fig. 2:**
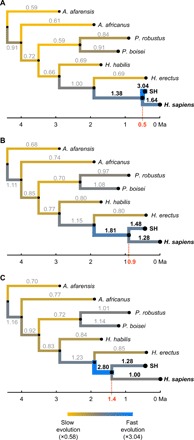
Branch-specific evolutionary rates obtained through the analysis of phylogeny-1. (**A**) Evolutionary rates obtained when setting the SH–modern human divergence time at 0.5 Ma ago. (**B**) Rates obtained when setting this divergence at 0.9 Ma ago, which is the scenario associated with the minimum SD of all the rates across the tree. (**C**) Rates obtained when setting divergence at 1.4 Ma ago. SH–modern human divergence times older than 1.4 Ma ago result in even higher rates for the branch antedating the SH–modern human separation, referred to in the following figures as the LCA branch. Evolutionary rates are provided above each branch (gray for rates that remain roughly constant in all scenarios, and black for rates associated with the Neanderthal–modern human clade, which are affected by changes in the SH–modern human divergence time).

**Fig. 3:**
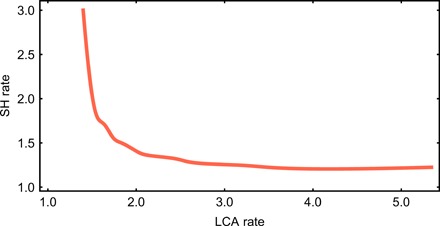
Relationship between the evolutionary rate at the SH branch and at the LCA branch. Relationship observed when analyzing the first phylogenetic scenario (phylogeny-1). Evolutionary rates at both branches show an inverse and nonlinear relationship such that very high rates at the SH branch are associated with very low rates at the LCA branch and vice versa. This effect can be visualized in Fig. 2, which shows how these rates change depending on the assumed SH–modern human divergence time.

Changing the divergence time between the SH and the modern human branches has strong effects on the length of the SH branch and the antedating branch, as well as on their associated evolutionary rates. Very late SH–modern human divergence times result in very short lengths for the SH branch, which result, in turn, in very fast evolutionary rates for this lineage. On the contrary, too early SH–modern human divergence times result in very short lengths for the phylogenetic branch leading to their LCA, which is reflected in a very high evolutionary rate for this branch. Figure 2 shows how the evolutionary rates associated with the Neanderthal–modern human clade (those corresponding to the SH branch, to the modern human branch, and to the LCA branch) differ substantially when modifying the SH–modern human divergence time as described above. Because the timing of the Neanderthal–modern human LCA is the only one allowed to change, there is an inverse relationship between the evolutionary rate of the branch leading to SH hominins (or to Neanderthals) and the branch subtending it, such that a slower rate in the SH branch is associated with a faster rate in the subtending branch (Fig. 3 and fig. S4).

**Fig. 4:**
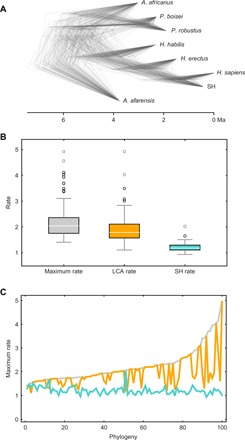
Variation of evolutionary rates obtained through the analysis of 100 trees. (**A**) Densitree showing the sample of 100 randomly selected trees used in the calculations. (**B**) Boxplot comparing the maximum evolutionary rate (gray), the LCA rate (orange), and the SH rate (teal) in the 100 phylogenies. (**C**) Evolutionary rates obtained in the analysis of each of the 100 phylogenies showing the maximum rate across the tree (gray), the LCA rate (orange), and the SH rate (teal). Phylogenies in (C) are sorted according to their maximum evolutionary rate. The plot shows that the LCA rate is the maximum rate in a majority of phylogenies (59 of 100), whereas the SH rate is the maximum rate only in three phylogenies. In all other cases, the maximum rate is found in other branches (in most cases, in the *P. boisei* branch).

**Fig. 5:**
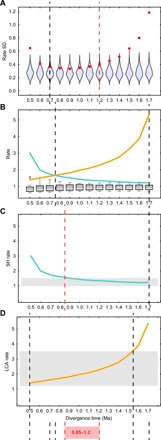
Most likely Neanderthal–modern human divergence time based on the analysis of phylogeny-1. (**A**) Comparison of observed SDs of all the rates across the hominin phylogeny (red points) with the distributions of SDs obtained when simulating evolution over the same tree at a constant rate. (**B**) Comparison of evolutionary rates at the SH branch (teal), LCA branch (orange), and all the other branches (gray) obtained for the different SH–modern human divergence times. (**C**) Comparison of the SH rate (teal line) with the 95% interval of rates obtained for this branch through the analysis of 100 phylogenies (gray box). (**D**) Comparison of the LCA rate (orange line) with the 95% interval obtained for that branch through the analysis of 100 phylogenies (gray box). Black dashed lines bracket the most likely divergence times according to each analysis. Red dashed lines indicate the minimum and maximum values obtained through all the analyses and bracket the most likely divergence time when all results are considered together. Equivalent results based on the analysis of phylogeny-2 are provided in fig. S5.

The analysis of 100 phylogenies yields very few cases (3 of 100) where the SH branch shows the highest rate across the complete tree, but a majority of cases (59 of 100) where the antedating branch shows the highest rate across the tree (Fig. 4). According to these results, scenarios with a divergence time older than 0.75 million years (Ma) ago, which result in the LCA branch showing the highest evolutionary rate, are more likely than scenarios with a younger divergence time (Fig. 5). The fact that the branch leading to the SH–modern human clade tends to show the highest evolutionary rate in most phylogenies shows that dental divergence was strongest in the later stages of the evolution of the genus *Homo*.

The null expectation that dental shape has evolved neutrally across the complete hominin phylogeny is accepted only if the Neanderthal–modern human divergence is within the 0.7- to 1.2-Ma interval (Fig. 5A and table S3), which strongly suggests against divergence times outside this interval. The expectation of neutral dental evolution is supported by previous studies (*19*) and was tested against simulated scenarios reflecting genetic drift and excluding selection (*27*). The standard deviation (SD) of the evolutionary rates across the tree reaches its minimum value at 0.9 Ma ago, although the tree SDs are low and very similar for the 0.7- to 1.1-Ma interval. Figure 5B shows that the rates corresponding to the SH branch and the subtending branch become equal when the divergence time is set at 0.7 to 0.8 Ma ago. Divergence times that are substantially younger or older than 0.75 Ma ago result in evolutionary rates for the SH branch or for the antedating branch that are extremely far from the range of variation observed for all the other branches (Fig. 5B). The evolutionary rate at the SH branch falls within the 95% interval calculated for that branch through the analysis of 100 phylogenies only when the Neanderthal–modern human divergence time is older than 0.8 Ma ago (Fig. 5C). The 95% interval of rates for the antedating branch is very broad, so most divergence times are compatible with the values calculated for this branch (Fig. 5D). The combined result of all these analyses yields an interval of 0.8 to 1.2 Ma ago as the most likely divergence time for the SH branch and the modern human branch and, therefore, for the Neanderthal and modern human lineages. Rerunning these analyses using the MCC tree calculated by Dembo and colleagues (*20*) provides even older divergence times, with a minimum divergence time of 0.9 Ma ago calculated from the combination of all the analyses (fig. S5 and table S4).

Assuming a Neanderthal–modern human divergence at approximately 600 ka ago, the age that the most recent molecular studies seem to point to (*16*, *17*, *22*), would have some consequences on the SH dental evolutionary rates. First, the SD of all the rates across the hominin phylogeny would show an unusually high value (although still within the obtained range), with respect to 1000 simulated neutral scenarios (*P* = 0.033 for phylogeny-1; see Fig. 5A and table S3). Second, assuming a divergence time of 600 ka ago would imply that the evolutionary rate at the SH branch was the highest across the hominin phylogeny (1.3 times greater than the evolutionary rate at the LCA branch). According to the analysis of 100 different phylogenies sampled from Dembo’s study (*20*), this scenario is not likely (Fig. 4). In addition, the evolutionary rate at the SH branch in a 600-ka divergence scenario would be 1.99, a value that is well outside the 95% interval of rates observed for the SH branch through the analysis of Dembo’s 100 trees (Fig. 5C). An evolutionary rate of 1.99 at the SH branch is lower than just one value observed in the analysis of 100 phylogenies (2.05), which is a clear outlier with respect to all rates observed at this branch (Fig. 4B). Results of the different analyses carried out in this study show that SH hominins must be separated by at least 400 ka from the Neanderthal–modern human LCA to maintain the evolutionary rate of SH hominins within the range of variation observed for other hominins. Therefore, making a ca. 600-ka divergence compatible with similar evolutionary rates between SH hominins and other hominin species would require a ca. 200 ka age for SH hominins, which is considerably younger than all the values that have been calculated for this population (*12*–*14*).

## DISCUSSION

Evolutionary rates measured in this study are heavily influenced by branch lengths, such that short branches accumulating strong dental changes result in high rates. Young divergence times between Neanderthals and modern humans result in short SH branches and, in turn, in the observed high evolutionary rates for the SH hominins. Therefore, if SH hominins were younger than 430 ka, then they would be compatible with divergence time between Neanderthals and modern humans postdating 800 ka ago without requiring exceedingly high evolutionary rates. More specifically, the ca. 600-ka divergence indicated by the most recent molecular estimates (*22*) would be compatible with average evolutionary rates for the SH sample if these hominins were as young as 200 ka. This scenario is worth considering because the age of SH hominins has been controversially discussed in the past (*2*, *12*, *13*). The most recent studies, however, based on luminescence and paleomagnetic analyses, securely point to an age of 430 ka for these fossils (*14*). This figure is further supported by genetic analyses dating the SH hominins to approximately 400 ka ago based on the length of its mtDNA branch, with a 95% highest posterior density interval of 150 to 650 ka (*15*). This interval is admittedly quite broad, and it implies that SH hominins can be younger than 430 ka. On the basis of these data, however, they can also be substantially older, which would necessarily push the Neanderthal–modern human divergence to an even older date. Additional evidence supporting a ca. 430 ka age for the SH sample comes from other molecular studies. Those studies demonstrate that SH hominins share the same mtDNA lineage as Denisovans, which differs from the Neanderthal and modern human mtDNA lineages (*15*). According to Posth and colleagues (*28*), the Denisovan-SH mtDNA lineage is the primitive one for the Neanderthal clade, and the classic Neanderthal mtDNA lineage was acquired posteriorly through an introgression event from modern humans that they date at 219 to 468 ka ago. If this model is correct, then the SH population has to predate this introgression event, which lends additional support to a >400 ka age for the SH sample. Therefore, on the basis of the current combined geochronological and molecular evidence, an age of about 430 ka for the SH hominins is the most reasonable assumption, so other explanations are required to account for the present results. Also related to branch lengths, it can be argued that the analytical approach presented in this study favors older Neanderthal–modern human divergence times because it assumes longer branch lengths (and, therefore, slower evolutionary rates) for other hominin species. This potential bias is accounted for by using 100 different phylogenetic scenarios based on Bayesian analyses of hominin phylogenetic relationships (*20*), some of which show branch lengths for the other species that are as short as the SH branch. Still, the analyses based on those 100 phylogenies also indicate that Neanderthal–modern human divergence times younger than 800 ka ago are very unlikely. This means that methodological artefacts are unlikely to drive the observed results, so biological factors are required to explain them.

A fast evolutionary rate in the early Neanderthal populations represented by SH hominins, which would be a necessary consequence of a Neanderthal–modern human divergence postdating 800 ka ago, can result from strong selection on dental shape in these hominins. Although this scenario is initially plausible, it is also very unlikely that the evolution of the early segment of the Neanderthal lineage was characterized by a fast dental evolution that is not observed in any other hominin species (*19*) (not even in those of the genus *Paranthropus*, which are characterized by an extreme degree of postcanine megadontia). This strong selection scenario is unlikely for two reasons. First, the dental shape differences observed in SH hominins with respect to an hypothetical ancestral morphology (*3*), and also with respect to more primitive configurations as those observed in *Homo erectus*, do not have a functional significance and are considered to be selectively neutral (*29*). Therefore, it is very unlikely that those dental variations were the target of the strong selection implied by unusually fast evolutionary rates. Second, the dentition of SH hominins is the only skeletal region that shows a highly derived state. Other traits related to mastication, such as facial and mandibular anatomy, show clear Neanderthal affinities in the SH hominins but not the hyper-derived Neanderthal state found in their dentitions (*10*), implying lower evolutionary rates. A strong selection scenario associated with some functional advantage would almost certainly involve other cranial regions apart from the teeth. The transitional state of most other traits in SH hominins most likely indicates that selection was not the major factor driving SH dental evolution.

As already mentioned, SH hominins show a dental anatomy that is not representative of the Neanderthal average but is substantially more derived. This observation, however, does not affect the design of this study nor its results. The study design does not require the SH dental anatomy to be representative of the broader Neanderthal range of variation. Rather, it is simply based on the fact that, whether representative or not, SH dental traits evolved from the same ancestral condition that classic Neanderthals evolved from and over the period of time that separates SH hominins for the Neanderthal–modern human LCA. Therefore, this study does not treat SH dental anatomy as representative of classic Neanderthals, but only as the dental shape that was characteristic of the SH population considering its evolutionary relationships and geological age. Considering this nonrepresentative and highly derived condition of the SH dentition, a plausible explanation for the fast dental evolution implied by a divergence postdating 800 ka ago points to SH dental anatomy as the result of a strong founder effect. In this scenario, ancestral populations to SH hominins would have had different dental morphologies, one of which would have been fixed in the SH sample because it was present in their direct ancestors. This scenario is, in theory, possible and might be supported by the geographical location of SH hominins on the Iberian Peninsula, where they may have been more isolated than other Neanderthal populations from mainland Europe. However, this scenario would imply that the SH dental phenotype was present, albeit in a small proportion, in the early Middle Pleistocene populations that SH hominins evolved from. Because of the scarcity of the fossil record, this scenario cannot be ruled out at the moment, but current fossil hypodigms do not show these derived dental configurations in any other hominins antedating the SH population, which undermines this hypothesis.

Another factor that may have potentially affected dental evolution in SH hominins is hybridization. On the basis of genetic analyses, it is now confirmed that hybridization happened between Neanderthals, modern humans, and Denisovans (*30*, *31*), probably quite often. Therefore, it can be safely assumed that different Middle Pleistocene hominin lineages hybridized when coming into contact. The high degree of mosaicism found in the SH population, with some traits showing a fully Neanderthal condition and others showing a much more primitive state, could potentially point to a hybrid origin. However, SH hominins do not show the skeletal anomalies that have been found in early-generation hybrids of living primate species, such as the presence of rotated or supernumerary teeth, and of sutural anomalies in the neurocranium and face (*32*). While a hybrid origin of SH hominins is certainly possible, this hypothesis does not have particularly strong support based on their anatomy nor on what we currently know about the phenotypic effects of hybridization.

The simplest explanation of the results presented in this study is that Neanderthals and modern humans diverged before 0.8 Ma ago, which would make evolutionary rates for the SH dentition roughly comparable to those found in other species. This divergence time is substantially older than the most recent aDNA-based estimates (*16*, *17*, *22*) but not so far off from previous estimates dating this divergence at ca. 800 ka ago (*24*). aDNA-based estimates of the divergence time between Neanderthals and modern humans differ substantially (*6*, *17*, *22*, *24*), indicating that a strict read of these values cannot drive the interpretations of the hominin fossil record. In addition, the divergence time obtained from the analysis of dental rates is strikingly similar to the divergence time of the SH-Denisovan and Neanderthal–modern human mtDNA lineages. The divergence between both mtDNA lineages has been estimated at ca. 1 Ma ago, with a 95% highest posterior density interval of 0.7 to 1.4 Ma ago (*15*). As explained above, the Neanderthal mtDNA lineage is thought to be the result of a relatively recent introgression event from modern humans (*28*). Therefore, the divergence time of the SH and modern human mtDNA lineages is much more accurately reflective of the Neanderthal–modern human population divergence than the divergence time between the Neanderthal and modern human mtDNA lineages, which reflects the maximum time for the introgression event and is substantially younger. The mtDNA divergence of SH hominins and modern humans, however, is still older than the population split time estimated from the nDNA between the modern human lineage and the Neanderthal-SH-Denisovan lineage, which has been recently calculated at 550 to 765 ka ago (*16*, *17*) or 520 to 630 ka ago (*22*). The mtDNA divergence time indicates the moment at which both mtDNA lineages started to accumulate mutations independently, whereas population split time represents the last time that both groups exchanged genetic material with each other through divergence and thus tends to be younger than mtDNA estimates. The results of the present study suggest that phenotypic differentiation in dental morphology started before the population split between the Neanderthal and the modern human lineages was complete. Although it is possible that these discrepancies result from the diverse methodological approaches used in different studies of genetic and phenotypic variation (*7*, *16*, *17*, *22*), it is also possible that these differences reflect diverging biological signals associated with different traits. In that case, the older time frame for phenotypic divergence suggested by dental variation will have deep implications for the way we interpret the hominin fossil record and the relationships between fossil specimens, particularly for those populations and time periods for which aDNA is not available.

If the phenotypic LCA of Neanderthals and modern humans was older than 800 ka, this would imply that all fossil hominins younger than this age are no longer valid candidates to occupy this ancestral position. Some fossils younger than this age, however, are frequently considered to be part of the last common ancestral species to Neanderthals and modern humans (*2*, *8*). These fossils, usually ascribed to *Homo heidelbergensis*, include European and African specimens, such as Mauer, Arago, Petralona, Bodo, Kabwe, etc., and maybe even some Asian specimens. If Neanderthals and modern humans diverged earlier than 800 ka ago, then all these fossils have to be related either to Neanderthals or to modern humans, or they can be part of a sister lineage to both of them. These fossils, however, cannot be ancestral to Neanderthals and modern humans because they would postdate their evolutionary divergence. An evolutionary relationship between these fossils and both Neanderthals and modern humans would be possible only if they were part of an older ancestral species that persisted in time as a relic species after the actual split of both lineages. Effectively, this scenario would mean that the *H. heidelbergensis* fossils are part of a sister group to Neanderthals and modern humans but that the evolutionary change from their putative ancestral populations did not involve speciation.

It has been suggested that the process of acquisition of a fully Neanderthal anatomy may have started earlier and may have been more gradual than the process of acquisition of a fully anatomically modern human configuration, which does not appear in the fossil record until ca. 200 ka ago (*4*). Incipient modern human traits are observed in the fossil record at ca. 300 ka ago (*33*), a figure that is in line with recent DNA-based estimates of modern human divergence at 260 to 350 ka ago (*18*). This contrasts with the observation of a fully Neanderthal (which can be even considered hyper-Neanderthal) dentition at 430 ka ago in the SH hominins. The discrepancies between the dates at which clear Neanderthal and modern human affinities are observed in the hominin fossil record may seem to indicate differential evolutionary rates in both lineages, which would affect the inferences made through the present study. However, they may simply reflect the incompleteness of the fossil record, particularly for the modern human lineage, as the SH sample is the only early Neanderthal population represented in the fossil record that shows such a derived dentition. New fossil findings, as well as the reassessment of previously known ones, are essential to shed more light on the process of acquisition of a fully anatomically modern human configuration.

## MATERIALS AND METHODS

### Experimental design

The major goal of this study was to measure evolutionary rates for dental shape in the earlier part of the evolution of the Neanderthal lineage and to compare them with the rates observed in other hominin species. The calculation of these evolutionary rates assumed different phylogenetic scenarios and different divergence times between Neanderthals and modern humans to determine the effect of these sources of uncertainty on the inferred rates. The results of these analyses have important implications regarding the mechanisms promoting dental evolution in early Neanderthals, the most likely divergence time between Neanderthals and modern humans, and, more generally, the interpretation of the Middle Pleistocene fossil record. The experimental design consisted of a three-step process including (i) the calculation of ancestral dental shapes at all the nodes of the hominin phylogeny using an mvBM approach (*25*), (ii) the calculation of the amount of change per branch as the difference between descendant and ancestral dental shapes, and (iii) the comparison of the observed amounts of change per branch with those expected when simulating evolution at a constant rate across all the branches of the phylogeny (*19*). The data and methodological approaches used in the study are explained in detail below.

### Data

Species-specific dental shape was calculated for eight hominin species for which data on the variation of all postcanine teeth (upper and lower premolars and molars) were available. This sample included *Australopithecus afarensis*, *Australopithecus africanus*, *Paranthropus robustus*, *Paranthropus boisei*, *Homo habilis* (including *H. habilis* and *Homo rudolfensis*), *H. erectus* (including only Asian specimens), SH sample (as representative of *H. neanderthalensis*), and *H. sapiens* (table S1). Classic Neanderthals were not included in the analyses because their exact relationship with the SH fossils (which can be directly ancestral or a sister group within the Neanderthal lineage) is currently unknown (*16*). The analysis of the SH fossils is deemed substantially more relevant than the analysis of classic Neanderthals because they are closer to the divergence point between the Neanderthal and the modern human lineages, thus allowing for a finer-detailed analysis. When classic Neanderthals are used, a divergence time of 500 ka ago yields an average evolutionary rate for the Neanderthal branch and results that generally agree with the expectation of similar evolutionary rates across all the branches of the hominin phylogeny (fig. S6) (*19*). A 500-ka divergence, however, is younger than the youngest bound provided by the most recent molecular and anatomical estimates, which indicates that fossils that are further from the Neanderthal–modern human divergence point do not provide enough resolution to time this divergence.

Specimens with a clear taxonomic affiliation with one of these eight groups were included in the analyses. Sample size for the different species differed substantially, ranging in most cases from 3 to 53 specimens per species and tooth position, with only three cases where sample size is smaller (table S2): M^2^ and M^3^ for *A. afarensis* (*n* = 2) and P_4_ for *A. africanus* (*n* = 1). Considering all teeth together, sample size ranged from 5 individuals represented by at least one tooth position (*P. robustus* and *P. boisei*) to 53 individuals represented by at least one tooth position (*H. sapiens*), with intermediate values for the other groups (table S2). This variation in sample sizes, however, is unlikely to affect results, as previous analyses based on jackknifing (reducing all sample sizes to *n* = 3) and bootstrapping have demonstrated that the constant evolutionary rates for dental shape in which the present study relies are very robust to sample size and composition (*19*). Shape variation was described using configurations of landmarks and semilandmarks placed on occlusal photographs of premolars and molars and that have been used in previous studies of hominin dental variation (fig. S1) (*3*, *19*). Procrustes superimposition (*34*) was used to remove non–shape variation corresponding to the position, size, and orientation of specimens. Procrustes superimposition was carried out for each tooth position separately, but information related to each tooth was later merged to study all postcanine variation together (*19*). A principal components (PC) analysis of Procrustes-superimposed coordinates was carried out, and PC scores were used in subsequent calculations. Variation in dental size was not considered because it is much more heterogenous than variation in dental shape, with some branches showing substantially faster rates than others (*19*). Dental traits are considered to be a good proxy for neutral genetic data because they tend to be highly heritable and selectively neutral (*29*).

### Phylogenies

The uncertainty about hominin phylogenetic relationships was addressed in different ways. First, two different phylogenetic scenarios were explored (fig. S3). The first one (phylogeny-1) is based on the first and last appearance dates of different hominin species (*35*), and it reflects the most broadly agreed hominin phylogenetic relationships (*19*, *36*). The second one (phylogeny-2) corresponds to the MCC tree obtained as part of a previously published Bayesian analysis of hominin phylogenetic relationships (*20*). This phylogeny was pruned to include only those species for which data on dental shape variation were available. The major differences between the first and the second phylogenetic scenarios concern the total length of the tree measured as the patristic distance (the sum of all the branches separating two given species) between the most basal node and the *H. sapiens* tip (approximately 4.5 Ma for phylogeny-1 and 6.2 Ma for phylogeny-2), the lengths of the different branches, and the phylogenetic position of *A. africanus*, which is placed as a sister group to all *Paranthropus* and *Homo* species in the first phylogeny (*19*) and only to *Paranthropus* in the second (*20*). The phylogenetic position of *A. africanus*, however, is unstable across different studies, with previous analyses setting it as a sister group only to *Homo* (*21*). On the basis of previous studies demonstrating an evolutionary relationship between Neanderthals and SH hominins (*16*), the phylogenetic branch leading to Neanderthals was replaced by the branch leading to SH. This was attained by changing the length of the Neanderthal branch so that it reflects a geological age for the SH sample of 430 ka (*14*).

Using these two phylogenies, the age of the Neanderthal–modern human LCA was changed from 500 ka to the age of the node separating *H. erectus* from the Neanderthal–modern human lineage (1.7 Ma in phylogeny-1 and 2.6 Ma in phylogeny-2) at 100-ka intervals. The ages of all the other nodes—and, consequently, the other branch lengths—were kept constant. Variation in evolutionary rates across all these different divergence time scenarios was assessed and compared with results based on the analysis of different phylogenetic topologies. The use of these different phylogenetic trees explicitly addressed phylogenetic uncertainty by recalculating evolutionary rates in a sample of 100 trees that were randomly selected out of a complete sample of 60,000 phylogenies generated in Dembo’s Bayesian analysis (*20*). This sample excluded phylogenies in which one or more branches had lengths shorter than 70 ka, which is the shortest possible length of the SH branch, obtained when the Neanderthal–modern human LCA is dated to 500 ka ago. The use of these different phylogenies addressed the uncertainty related to unclear phylogenetic relationships and branch lengths. As for the former, different phylogenies recover different evolutionary relationships across species. As for the latter, branch lengths differ in all the different trees. Therefore, although Dembo and colleagues’ study did not specifically model the uncertainty due to the age of each fossil species, that uncertainty is implicitly included in the calculations due to the different branch lengths recovered in their sample of trees. Evolutionary rates were calculated over this sample of 100 trees, and ranges of variation were compared with results obtained when analyzing the two previously described phylogenetic contexts. Possible hybridization events between lineages were not included in these calculations.

### Statistical analysis: Ancestors and evolutionary rates

Ancestral values at the different nodes of the hominin phylogeny were calculated using an mvBM approach (*25*), which relaxes the assumption that different branches have evolved at a constant rate following a standard Brownian motion (BM) model. Biologically, this approach accounts for the fact that ancestors may have not been intermediate in shape between their descendent lineages, but more similar to one of the descendant groups (*4*). This situation would be reflected in different evolutionary rates across the tree, with some branches showing stasis and others showing fast evolution. Through simulations, an mvBM approach has been demonstrated to produce results equivalent to standard BM under standard BM conditions and to substantially outperform standard BM approaches when evolutionary bursts (very high evolutionary rates over short periods of time) occur (*37*). In addition, the results of this study indicate that standard BM approaches (*38*) do not accurately recover differential evolutionary rates that result from changing branch lengths, particularly for very early divergence times, as very similar SDs of rates are obtained when varying divergence times (tables S3 and S4). Short branches are expected to show fast evolutionary rates because they accumulate phenotypic change over a very short period of time. Therefore, the results obtained from standard BM approaches are counterintuitive because they yield similar evolutionary rates regardless of branch length (table S3). As inferred from these results, standard BM approaches do not accurately recover evolutionary bursts that are restricted to single branches, but they distribute change across the neighboring branches. Ancestral values were calculated using species-specific PC scores with the R package evomap (*39*). All PC scores were included in the calculations, and they were later transformed to ancestral landmark coordinates. Procrustes distances between descendant and estimated ancestral morphologies were compared with Procrustes distances between descendant species and ancestors obtained when simulating evolution at a constant rate across the whole hominin phylogeny 1000 times (*27*). For these simulations, a per-generation variance rate was calculated on the basis of available data using a generalized least squares (GLS) approach (*38*). These calculations were carried out using the packages Morphometrics (*40*) and Phylogenetics (*41*) for Mathematica and followed a transformation of the hominin phylogenetic tree to generations using a constant generation time of 25 years. For each branch, a ratio was calculated between the observed amount of change and the corresponding simulated amount of change in the neutral scenario where all the species were evolving at the same rate. Ratios lower than 1 indicate branches that are evolving slowly and undergoing stasis, whereas ratios greater than 1 indicate fast evolution and, when very high, are likely indicative of directional selection (*19*). For the sake of simplicity, this ratio of observed to simulated change per branch is referred to throughout the text as rate, but these are not rates in the strict sense because they do not represent change per unit of time.

Results obtained from the calculation of evolutionary rates when assuming different divergence times for Neanderthals and modern humans were compared in different ways. Some of these comparisons involved rates across the complete tree, whereas others focused on the branches directly related to the Neanderthal–modern human divergence. For the former, the SDs of all rates in each tree were compared with those simulated in the constant rate scenarios, and *P* values were calculated as the proportion of simulated SDs exceeding the observed SD for each divergence time. For the latter, the evolutionary rates of the SH branch and subtending branch (LCA branch) were compared to each other and to the rates observed in the other branches, as well as to the corresponding rates obtained when analyzing 100 different phylogenetic topologies. These diverse comparisons provided different age intervals for the Neanderthal–modern human LCA. The overlapping region of these different estimates is considered the most likely divergence time between Neanderthals and modern humans, and the lower bound of the interval is interpreted as the minimum age of their LCA.

## Supplementary Material

http://advances.sciencemag.org/cgi/content/full/5/5/eaaw1268/DC1

Download PDF

## SUPPLEMENTARY MATERIALS

Supplementary material for this article is available at http://advances.sciencemag.org/cgi/content/full/5/5/eaaw1268/DC1 Fig. S1. Configurations of landmarks and semilandmarks used to describe the shape of posterior teeth. Fig. S2. Principal components analysis of dental shape in hominins. Fig. S3. Comparison between the two phylogenetic scenarios used in this study. Fig. S4. Relationship between the evolutionary rate at the SH branch and at the LCA branch in phylogeny-2. Fig. S5. Most likely Neanderthal–modern human divergence time obtained from the analysis of Dembo and colleagues’ MCC tree (phylogeny-2). Fig. S6. Rate analysis based on classic Neanderthals and phylogeny-1. Table S1. List of specimens used in this study. Table S2. Sample size per species and tooth position. Table S3. Comparison of observed and simulated SDs of rates across the tree for the different SH–modern human divergence times. Table S4. Comparison of observed and simulated SDs of rates across the tree for the different SH–modern human divergence times calculated when using the Dembo *et al*. phylogenetic tree (phylogeny-2). References (*42*–*46*)
