# Supplementary material for: Dental evolutionary rates and its implications for the Neanderthal–modern human divergence
Source: Sci Adv. 2019 May 15;5(5):eaaw1268. doi: 10.1126/sciadv.aaw1268 (PMC6520022; doi:10.1126/sciadv.aaw1268)
Supplement: http://advances.sciencemag.org/cgi/content/full/5/5/eaaw1268/DC1 [file supp_5_5_eaaw1268__index.html]

Science Advances | Science Advances

## Supplementary Materials

**This PDF file includes:**

- Fig. S1. Configurations of landmarks and semilandmarks used to describe the shape of posterior teeth.
- Fig. S2. Principal components analysis of dental shape in hominins.
- Fig. S3. Comparison between the two phylogenetic scenarios used in this study.
- Fig. S4. Relationship between the evolutionary rate at the SH branch and at the LCA branch in phylogeny-2.
- Fig. S5. Most likely Neanderthal–modern human divergence time obtained from the analysis of Dembo and colleagues’ MCC tree (phylogeny-2).
- Fig. S6. Rate analysis based on classic Neanderthals and phylogeny-1.
- Table S1. List of specimens used in this study.
- Table S2. Sample size per species and tooth position.
- Table S3. Comparison of observed and simulated SDs of rates across the tree for the different SH–modern human divergence times.
- Table S4. Comparison of observed and simulated SDs of rates across the tree for the different SH–modern human divergence times calculated when using the Dembo *et al*. phylogenetic tree (phylogeny-2).
- References (*42*–*46*)

Download PDF

**Files in this Data Supplement:**

- Adobe PDF - aaw1268\_SM.pdf
