# Supplementary material for: Dental evolutionary rates and its implications for the Neanderthal–modern human divergence
Source: Sci Adv. 2019 May 15;5(5):eaaw1268. doi: 10.1126/sciadv.aaw1268 (PMC6520022; doi:10.1126/sciadv.aaw1268)
Supplement: Download PDF [file aaw1268_SM.pdf]

Science Advances
AAAS

advances.sciencemag.org/cgi/content/full/5/5/eaaw1268/DC1

Supplementary Materials for

Dental evolutionary rates and its implications for the Neanderthal–modern human divergence

Aida Gómez-Robles*

*Corresponding author. Email: a.gomez-robles@ucl.ac.uk

Published 15 May 2019, *Sci. Adv.* **5**, eaaw1268 (2019)
DOI: 10.1126/sciadv.aaw1268

This PDF file includes:

Fig. S1. Configurations of landmarks and semilandmarks used to describe the shape of posterior teeth.
Fig. S2. Principal components analysis of dental shape in hominins.
Fig. S3. Comparison between the two phylogenetic scenarios used in this study.
Fig. S4. Relationship between the evolutionary rate at the SH branch and at the LCA branch in phylogeny-2.
Fig. S5. Most likely Neanderthal–modern human divergence time obtained from the analysis of Dembo and colleagues' MCC tree (phylogeny-2).
Fig. S6. Rate analysis based on classic Neanderthals and phylogeny-1.
Table S1. List of specimens used in this study.
Table S2. Sample size per species and tooth position.
Table S3. Comparison of observed and simulated SDs of rates across the tree for the different SH–modern human divergence times.
Table S4. Comparison of observed and simulated SDs of rates across the tree for the different SH–modern human divergence times calculated when using the Dembo *et al*. phylogenetic tree (phylogeny-2).
References (*42–46*)

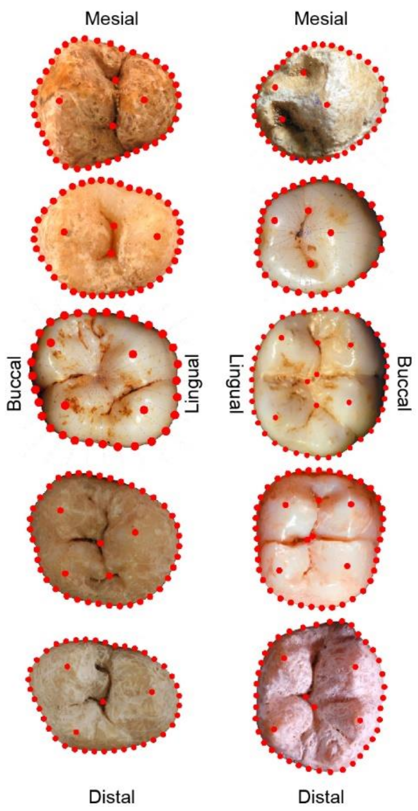

Fig. S1. Configurations of landmarks and semilandmarks used to describe the shape of posterior teeth. Upper teeth are shown on the left and lower teeth on the right, following the sequence P3, P4, M1, M2, M3 from top to bottom. Landmark definitions follow refs. (*11*, *42*–*45*). Photo credit: Aida Gómez-Robles, photographs taken at University of Granada.

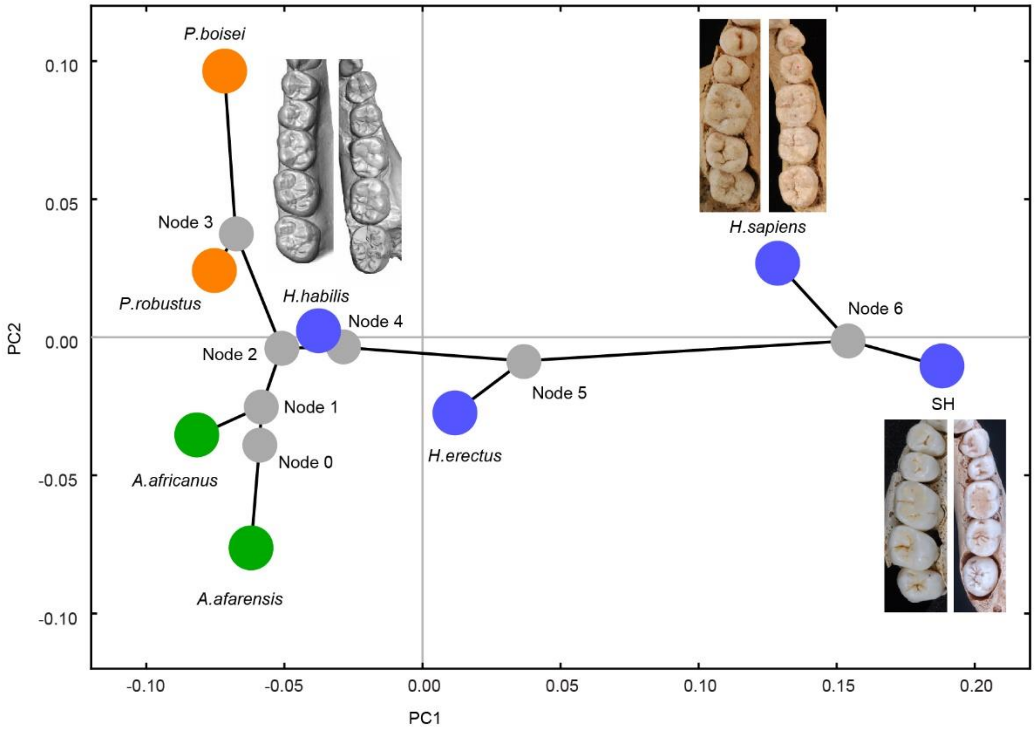

Fig. S2. Principal components analysis of dental shape in hominins. *Australopithecus* species are represented in green, *Paranthropus* in orange, *Homo* in blue, and nodes in gray. Examples are provided of representative posterior dentitions of *Homo habilis*, *Homo sapiens* and Sima de los Huesos hominins. Images of *H. habilis* teeth are from ref. (*46*) and correspond to KNM-ER 1802 (lower teeth) and a reconstruction of the upper arcade based on the isolated teeth of KNM-ER 1590, with the M2 duplicated as a surrogate for the lacking M3. Photo credit: Ana Muela Pareja and Aida Gómez-Robles, photographs taken at Institute of Health Carlos III and University of Granada.

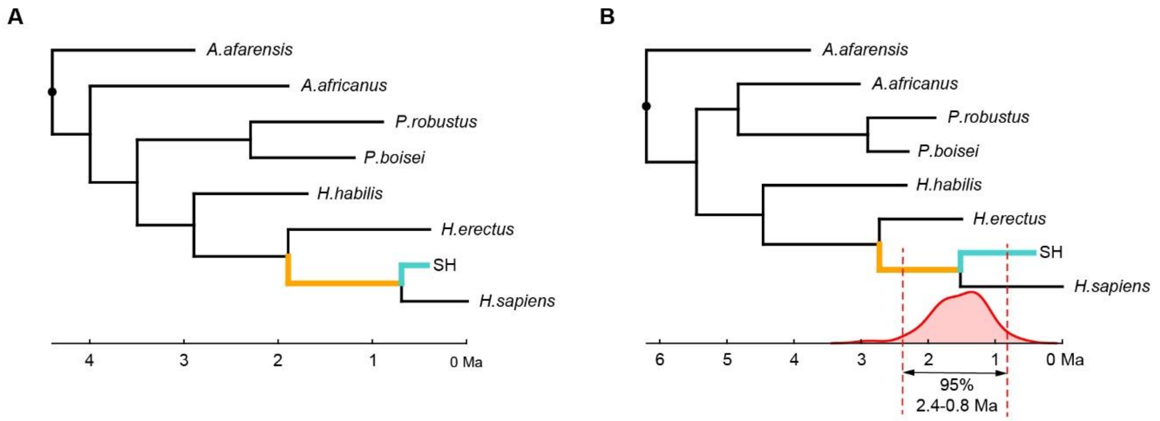

Fig. S3. Comparison between the two phylogenetic scenarios used in this study. (**A**) Phylogeny-1: Phylogeny used in ref. (*19*), with the SH branch highlighted in teal and the LCA branch highlighted in orange. (**B**) Phylogeny-2: MCC tree generated by a Bayesian analysis of hominin phylogenetic relationships (*20*). The distribution of Neanderthal-modern human divergence times obtained in the 60,000 phylogenies generated in Dembo and colleagues' analysis is provided underneath their MCC tree (represented in B). That distribution has a 95% interval of 0.8 to 2.4 Ma, with a median value of 1.5 Ma.

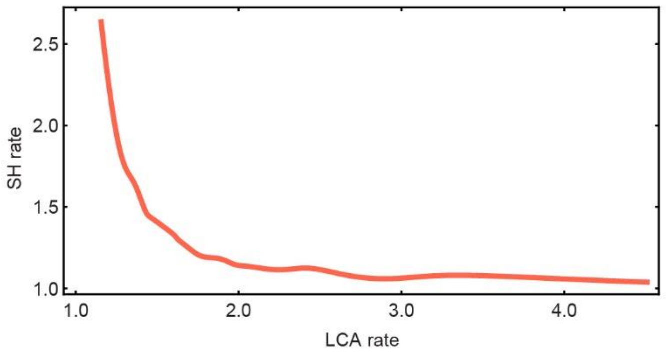

Fig. S4. Relationship between the evolutionary rate at the SH branch and at the LCA branch in phylogeny-2.

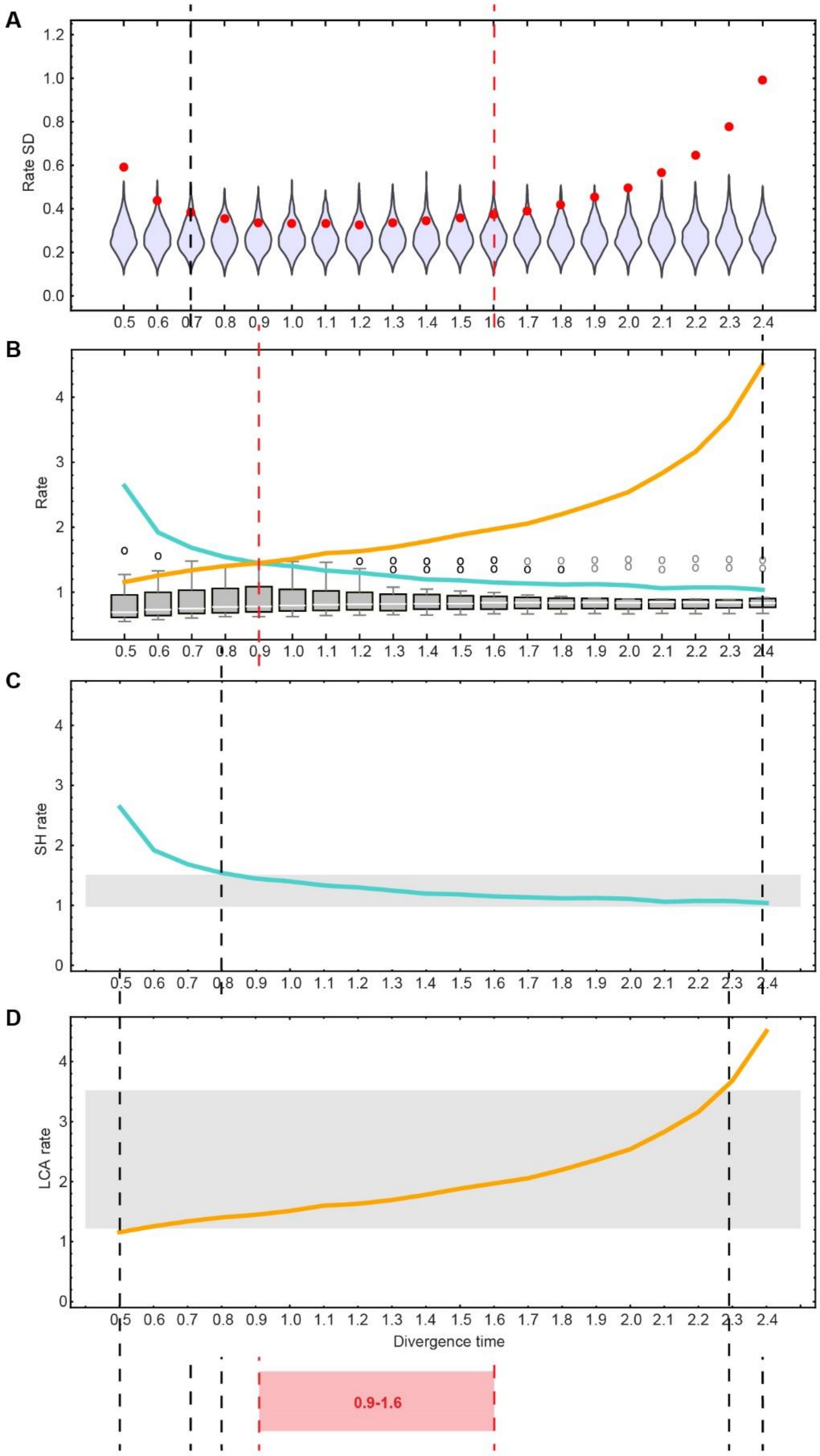

A
Rate SD
B
Rate
C
SH rate
D
LCA rate
Divergence time
0.9-1.6

Fig. S5. Most likely Neanderthal–modern human divergence time obtained from the analysis of Dembo and colleagues' MCC tree (phylogeny-2). (**A**) Comparison of observed SDs of all the rates across the hominin phylogeny (red points) with the distributions of SDs obtained when simulating evolution over the same tree at a constant rate. (**B**) Comparison of evolutionary rates at the SH branch (teal), LCA branch (orange) and all the other branches (gray) obtained for the different SH-modern human divergence times. (**C**) Comparison of the SH rate (teal line) with the 95% interval of rates obtained for this branch through the analysis of 100 phylogenies (gray box). (**D**) Comparison of the LCA rate (orange line) with the 95% interval obtained for that branch through the analysis of 100 phylogenies (gray box). Discontinuous lines bracket the most likely divergence times according to each analysis. Red discontinuous lines indicate the minimum and maximum values obtained through all the analyses, and bracket the most likely divergence time when all results are considered together.

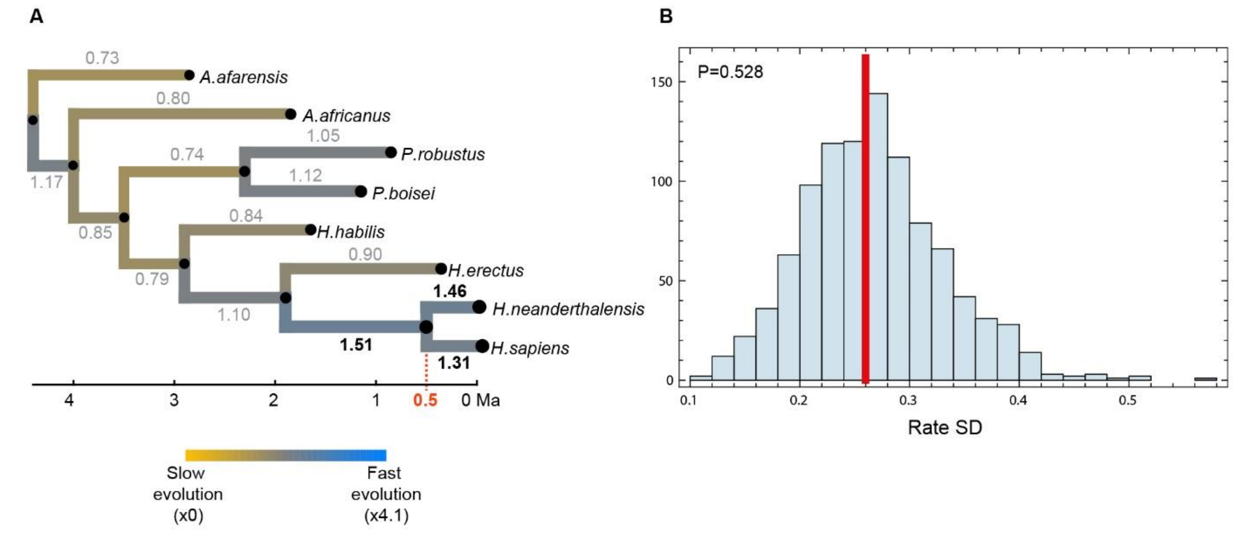

Fig. S6. Rate analysis based on classic Neanderthals and phylogeny-1. (**A**) Phylogeny-1 showing dental evolutionary rates obtained when using classic Neanderthals instead of SH hominins and assuming a Neanderthal-modern human divergence of 0.5 Ma. (**B**) Comparison of the observed standard deviation of all the rates across the tree (red vertical line) with the distribution of standard deviations obtained when simulating evolution of dental shape over the same phylogeny at a constant rate 1000 times. Results are compatible with a 500 ka divergence between Neanderthals and modern humans even if this is implausible based on the most recent aDNA-based estimates (*22*). This indicates that classic Neanderthals do not provide enough resolution to time the Neanderthal-modern human divergence because they are too far this divergence point. Data included in the figure are from reference (*19*).

Table S1. List of specimens used in this study.

Species	Teeth
A. afarensis	A.L. 128-23, A.L. 145-35, A.L. 188-1, A.L. 199-1, A.L. 200-1a, A.L. 207-13, A.L. 266-1, A.L. 288-1, A.L. 333-1, A.L. 333-2, A.L. 333W-57, A.L. 333W-60, A.L. 400-1a, A.L. 486-1, LH 2, LH 3, LH 4, LH 6, LH 17, LH 21
A. africanus	MLD 2, MLD 4, MLD 6, MLD 18, MLD 19, MLD 23, MLD 28, Sts 1, Sts 8, Sts 17, Sts 21, Sts 22, Sts 24, Sts 52, Sts 56, Sts 57, Stw 2, Stw 3, Stw 6, Stw 14, Stw 73, Stw 183, Stw 252, Stw 384, Stw 498, TM 1511, Taung
P. robustus	SK 11, SK 13/14, SK 23, SK 46, SK 48, SK 55, SKW 5, SKW 10, SKX 4446, TM 1517
P. boisei s. l.	KNM-CH 1, KNM-ER 729, KNM-ER 3230, KNM-ER 15930, KMN-WT 17400, Omo L51-79, Omo L338X-40, Omo L427-7, OH 5, Peninj 1
H. habilis s. l.	KNM-ER 1590, KNM-ER 1802, KNM-ER 1805, KNM-ER 1813, OH 4, OH 6, OH 7, OH 13, OH 16, OH 21, OH 24, OH 39, OH 41, OH 44, SE 255, Stw 53, Stw 80[a], Stw 151[a], SK 27
H. erectus	Sangiran 1a, 1b, 4, 5, 6, 7[b], Trinil 5, Zhoukoudian 19, 20, 25, 28, 29, 35.77, 36, 40, 41, 43, 44, 45, 46, 51, 52, 80, 90, 131, 137, 140, A2, B3.9, F1.5, G1.6, K1.96, PA327
**Sima de los Huesos[c]**	Atapuerca-Sima de los Huesos individuals I-XXVIII plus isolated teeth
***H. sapiens*[c]**	Abri Pataud, Almonda, Dolni Vestonice, Grimaldi, Isturitz, Les Rois, Mladec, Qafzeh, St Germain-La Riviere, Trou Magritte, AMNH (USA, but collection of European origin), UGR (Spain), CENIEH (Spain)

[a] Stw 80 and Stw 151 are also considered part of the *A. africanus* hypodigm.

[b] Including all isolated teeth in the Sangiran 7 sample.

[c] Sima de los Huesos and *H. sapiens* datasets are listed by site due to the large number of isolated teeth. All the other species are listed by specimen.

Table S2. Sample size per species and tooth position.

Species	P^3	P^4	M^1	M^2	M^3	P_3	P_4	M_1	M_2	M^3	Total[a]	Maximum
A. afarensis	4	3	6	2	2	7	8	6	9	7	54	9
A. africanus	7	7	9	9	7	4	1	4	4	8	60	9
P. robustus	5	5	5	4	4	3	4	4	4	3	41	5
P. boisei	3	3	3	3	3	4	5	4	5	4	37	5
H. habilis	4	5	11	7	6	4	4	5	4	3	53	11
H. erectus	8	6	7	7	8	10	5	11	11	5	78	11
**Sima de los Huesos**	15	14	13	16	19	15	19	17	17	19	164	19
H. sapiens	46	42	53	45	37	53	44	47	41	36	444	53

[a] Total values are not representative of actual sample sizes because in most cases they include several teeth belonging to the same individual (e.g., M^1, M^2 and M^3 of one single individual). Therefore, maximum values are more accurately reflective of sample sizes.

Table S3. Comparison of observed and simulated SDs of rates across the tree for the different SH–modern human divergence times. Results are based on the analysis of phylogeny-1.

		mvBM		Standard BM	
**Divergence time (Ma)**	**Simulated SD (range)**	**Observed SD**	**P-value**	**Observed SD**	**P-value**
**0.5**	0.11-0.60	0.66	**<0.001**	0.44	**0.025**
**0.6**	0.11-0.54	0.42	**0.033**	0.38	0.081
**0.7**	0.10-0.62	0.36	0.125	0.33	0.217
**0.8**	0.11-0.65	0.35	0.164	0.31	0.299
**0.9**	0.11-0.61	0.33	0.232	0.28	0.443
**1.0**	0.11-0.56	0.35	0.154	0.27	0.543
**1.1**	0.12-0.60	0.37	0.117	0.26	0.567
**1.2**	0.09-0.67	0.40	0.080	0.25	0.638
**1.3**	0.10-0.56	0.46	**0.018**	0.26	0.584
**1.4**	0.12-0.74	0.53	**0.008**	0.26	0.561
**1.5**	0.10-0.62	0.63	**<0.001**	0.26	0.580
**1.6**	0.10-0.57	0.82	**<0.001**	0.27	0.541
**1.7**	0.11-0.62	1.20	**<0.001**	0.28	0.510

Bold: divergence times that are incompatible with the null hypothesis of neutral evolution for dental shape across all hominin species.

Results obtained when using a mvBM approach are compared with those obtained when using standard BM approaches. Note that standard BM does not detect high standard deviations (resulting from unusually high evolutionary rates) associated with very early Neanderthal-modern human divergence times.

Table S4. Comparison of observed and simulated SDs of rates across the tree for the different SH–modern human divergence times calculated when using the Dembo *et al.* phylogenetic tree (phylogeny-2).

		mvBM		Standard BM	
**Divergence time (Ma)**	**Simulated SD (range)**	**Observed SD**	**P-value**	**Observed SD**	**P-value**
**0.5**	0.11-0.68	0.59	**0.001**	0.47	**0.010**
**0.6**	0.11-0.52	0.44	**0.018**	0.42	**0.028**
**0.7**	0.10-0.55	0.39	0.061	0.38	0.061
**0.8**	0.10-0.48	0.36	0.107	0.35	0.132
**0.9**	0.10-0.57	0.34	0.141	0.32	0.187
**1.0**	0.12-0.53	0.33	0.173	0.32	0.225
**1.1**	0.11-0.51	0.33	0.170	0.31	0.248
**1.2**	0.12-0.51	0.33	0.186	0.30	0.290
**1.3**	0.12-0.54	0.34	0.162	0.30	0.302
**1.4**	0.09-0.56	0.35	0.126	0.29	0.362
**1.5**	0.12-0.53	0.36	0.117	0.29	0.373
**1.6**	0.09-0.57	0.38	0.078	0.29	0.374
**1.7**	0.10-0.51	0.39	**0.044**	0.29	0.360
**1.8**	0.11-0.50	0.42	**0.026**	0.29	0.357
**1.9**	0.12-0.55	0.45	**0.009**	0.29	0.372
**2.0**	0.11-0.56	0.50	**0.003**	0.30	0.318
**2.1**	0.09-0.53	0.57	**<0.001**	0.30	0.342
**2.2**	0.11-0.51	0.64	**<0.001**	0.30	0.327
**2.3**	0.12-0.61	0.78	**<0.001**	0.30	0.336
**2.4**	0.10-0.63	0.99	**<0.001**	0.30	0.346

Bold: divergence times that are incompatible with the null hypothesis of neutral evolution for dental shape across all hominin species.

Results obtained when using a mvBM approach are compared with those obtained when using standard BM approaches. Note that standard BM does not detect high standard deviations (resulting from unusually high evolutionary rates) associated with very early Neanderthal-modern human divergence times.
